# Potash in Stomatitis

**Published:** 1853-11

**Authors:** J. H. Babington


### *Efficacy of Potash in Ulcerated Stomatitis and Cancrum Oris.*

BY J. H. BABINGTON, M. B.

On reading Dr. Duncan's practical paper, in the last Number of the *Dublin Quarterly,*' on the use of mercury in cancrum oris (a mode of treatment of which I do not question the efficacy), my recollection was called to an epidemic of ulcerative stomatitis. that occurred in the Coleraine Union Workhouse in 1849, in which I used chlorate of potash with great success.

The sympoms were as follows:—The lips, gums, and lining memmembrane of the cheeks, red, swollen, and tender to the touch; tongue swollen and indented by the teeth, covered over the surface with a foul tenatious coating, and in some places ash-colored sloughs. There was great pain when the cheeks were moved, touched, or any food introduced into the mouth, together with considerable general fever, and the secretions vitiated,

The first morning I observed the disease, two only complained; next day three more cases were presented; and on the fourth morning I had fifteen on the sick list, all from am ngst the boys in the workhouse.

The treatment adopted was a mild aperient of rhubarb and magnesia, and the administration of chlorate of potash, dissolved in water sweetened with syrup, in doses of four grains every fourth hour; the mouth was also washed with a weak lotion of solution of chloride of soda. They all recovered in about six days. I treated one case with alteratives and tonics, and it was three weeks before it got well; thereby proving the efficacy of the clorate of potash. [Here is no novelty; the medicine has been long given in such cases, and was first recommended by Mr. Hunt.]—*Rankin's Abstract.*

